# The Jewish doctors involved in the development of health resorts in eastern Galicia at the late 19th and early 20th century (Central and Eastern Europe)

**DOI:** 10.1007/s00508-018-1401-5

**Published:** 2018-11-07

**Authors:** Izabela Spielvogel, Krzysztof Spałek, Jarosław Proćków

**Affiliations:** 1 Department of Physiotherapy, Institute of Physiotherapy, Opole University of Technology, Prószkowska 76, 45-758 Opole, Poland; 2 Institute of Biology, University of Opole, Oleska 22, 45-052 Opole, Poland; 3 Institute of Biology, Faculty of Biology and Animal Science, Wrocław University of Environmental and Life Sciences, Kożuchowska 5b, 51-631 Wrocław, Poland

**Keywords:** Jewish doctors, Health resort medicine, History of medicine, Eastern Galicia, Central and Eastern Europe

## Abstract

**Background:**

The involvement of Jewish doctors and scientists in the development of health resorts in eastern Galicia (part of the Austrian monarchy after 1772, and since 1918 as part of independent Poland, now part of Ukraine) is unquestionable; however, awareness of this fact is not that common. Meanwhile, also due to their work and activity, small borderland resorts became important medical, cultural and social centers of the region. The involvement of Jewish doctors in the development of Galician health resorts resulted from, among others, the rich and multi-layered tradition and integration of Judaism with the hygiene regulations and moral principles of the religion. The eastern Galicia health resort culture, architecture, style of how free time was spent, along with treatment and disease prevention, contributed to a European identity in the region.

**Aim:**

This article constitutes an attempt to restore the memory of places and people who created the phenomenon of Galician health resorts, an important social amenity and whose contribution to this process is completely forgotten or omitted. Most of the physicians mentioned in this article died in concentration camps or were murdered by the Nazis.

**Methods:**

The article is based on the analysis of source texts drawn up in the German and Polish languages, including biographical archives, books, scientific articles, ego documents and press reports from the decades before WW I.

**Results:**

On the European level, eastern Galicia resorts were new resorts and the development took place mainly around 1900. The Jewish community constitutively contributed to the medical, economic and cultural development of the resorts. Its role in creating a resort culture is related both to the activity of the Jewish intelligentsia and wealthy bourgeoisie, as well as local craftsmen, tavern owners or shopkeepers. It resulted from a wealthy and multi-layered Jewish tradition and the integration of Judaism with the orders of hygiene and moral principles of religion.

## Introduction

Up to 1939 the Jewish population of Galicia constituted a significant percentage of the inhabitants of this region [1]. In 1918 Jews constituted approximately 10% of the inhabitants of Galicia. This region was inhabited by over 66% of all Jews in the Austro-Hungarian Empire [2, 3]. The development of the health resorts of eastern Galicia as part of the Austrian monarchy after 1772, and since 1918 as part of independent Poland, took place in the late nineteenth and early twentieth century. The wave of medical discoveries in balneoclimatology resulted in the beginning of organized therapeutic activities in health resort municipalities and climatic facilities, such as Morszyn, Delatyn, Druskieniki, Zaleszczyki, Jaremcze, Żabie, Mikuliczyn, Worochta and Truskawiec. The implementation of a medical system for therapy control was the basis of the success of Central European resorts. Within the territory of the Austrian monarchy, this was done in accordance with the state act on organization of public health services from 30 April 1870, and various regulations that were later implemented in individual crown countries (these were two Acts in Galicia from 1891 and 1908). Under the legal provisions, health resorts had to have a medical directorate and the therapy provided in the health resort must have been subjected to medical scientific management. Around 1900, the health resorts of eastern Galicia attracted the Jewish population. At the end of the nineteenth century, for example in Birštonas (Birsztany), the poor visitors of the health resort constituted a major part of the population visiting this municipality [4]. In the town two Jewish physicians had practices; however, large, international resorts that offered metropolitan anonymity enjoyed the greatest attention of Jewish visitors. Small borderland resorts were less visited, as Jews, especially Orthodox Jews, often experienced anti-Semitism there [5]. This was caused by many factors of which two were most important: integration of hygiene regulations with religious tradition, as Judaism integrated moral principles of religion, hygiene regulations and medicine into a coherent system, ordering, among others, health and disease prevention [6]. The second reason was related to the difficulties Jewish doctors faced to obtain specialization at that time in different fields of medicine [7]. To be able to develop and promote themselves professionally they chose new niches in medical majors, such as hygiene, laryngology or physical medicine, which constituted the basis of therapy at a health resort.

## Jewish doctors from Vienna and their concept of health resort treatment

In the nineteenth century, the University of Vienna or *Wiener Medizinische Schule* was primarily responsible for the development of physical medicine [7]. Therefore, with respect to the discussed issue, it is impossible to disregard the links between borderland health resorts and this scientific center. Vienna was also the place where the influences of Jewish doctors were very strong, as proved in the Polish prewar press note:

Two professions are becoming more influenced by the Jewish: medicine and advocacy. Fortunately, our medicine is still the most Polish-Christian and protected for a long time from Jews. In other Austrian provinces, such as in Vienna, the Jews have completely taken over medicine. Effects are terrible (translated by Izabela Spielvogel; [8]).

According to Wolfgang Krauss, the percentage of physicians in Vienna in the late nineteenth and early twentieth century amounted to over 70% of the total number [9]. In his studies on the development of physical medicine, Wilfried Teicher from Munich created a collective portrait of professors of medicine of Jewish origin in the German language area, which was arranged in a very characteristic scheme of three generations. These professors were mainly the sons of free practicing doctors and grandchildren of merchants or entrepreneurs. The high material situation was also an important element of the collective portrait, as it provided Jewish physicians with financial independence. Therefore, they obtained much better results in doctorate degrees or in the field of innovation [9]. Prof. Josef Seegen (1822–1904) was a Viennese researcher of Jewish origin whose activity reached the scope beyond the local scale in terms of the development of health resort culture, thus it also concerned the borderlands. The balneologist and diabetologist, a health resort doctor in Karlovy Vary (German Karlsbad), (1853–1884) was born in a merchant family in Polna in the Czech Republic. He studied in Prague and Vienna. Between 1857 and 1858, he published a two volume work entitled *Handbuch der allgemeinen und speciellen Heilquellenlehre*, which is considered canonical in the field of balneology. In 1859, at the University of Vienna, he was the first to lecture in the field of balneology. He was a cofounder of the first association promoting balneology and health resort medicine, the*Verein für Heilquellenkunde in Ősterreich.* Prof. Wilhelm Winternitz (1835–1917) was another Jewish researcher whose activity was related to Vienna. He was born in Josefov (German Josefstadt) in the Czech Republic. He studied in Prague and Vienna. He later practiced, for example in Gräfenberg (current Jaseniki in Czech Republic), where under the supervision of Dr. Joseph Schindler, a student of the well-known hydrotherapist, Vincenz Priessnitz (1799–1851), he learned about indications, contraindications and methodology of hydrotherapeutic treatment. In 1865, he obtained the first degree in the history of medicine hydrotherapy habilitation based on the work *Zur rationellen Begründung einiger hydrotherapeutischer Verfahren*. In the same year, he opened a private hydrotherapy facility in Kaltenleutgeben near Vienna [10]. In 1881 Winternitz became a professor at the University of Vienna, where he headed the first hydropathology department in the world. It should be emphasized that the matter was important as at the end of the nineteenth century, every European health resort had hydrotherapeutic premises where one of the three most popular hydrotherapy methods were used: methods of Wilhelm Winternitz, Sebastian Kneipp (1821–1897) or Vincenz Priessnitz (1799–1851) [11]. Enoch Heinrich Kisch (1841–1918) was another doctor, whose activity had a significant impact on the health-resort culture of the former Borderlands of Poland [7]. His brother, Alexander, was a rabbi in Prague, and his father, Josef was the founder of the first Jewish school in this city. Enoch Heinrich Kisch graduated from junior high school and medical studies at the University of Prague. After the doctorate degree, he was supported by a balneologist, Prof. Josef von Lösner (1809–1888) who helped him become a health resort doctor in 1863 in the Marianskie Lazne (German Marienbad) health resort. He opened a rational discourse in the medical community on the clinical and physiological foundations of balneoclimatic therapy. From 1868, he was also the editor of a prestigious scientific balneological journal *Allgemeine Balneologische Zeitung*, in which all the world’s prominent scientists published their works regarding health resort therapy and related fields. Thanks to his activities, he especially contributed to the development of a model of modern health resort treatment and balneology; he also popularized this field among patients and increased their pro-health awareness with respect to lifestyle diseases.

## Jewish doctors from Cracow and Lvov and their involvement in development of physical medicine at health resorts

Apart from the facility in Vienna, borderland resorts were associated with native universities, among which the universities in Cracow and Lvov prevailed. Until 1900 at the Jagiellonian University, the largest number of people of Jewish origin received a doctorate degree in the legal and medical faculties. The Jews also constituted 30% of PhDs in medicine or “maybe more, as many of them changed their typical Jewish-German names” (translated by Izabela Spielvogel; [12]). Considering that they constituted 1/10 of the population of Galicia at that time, the proportions in relation to the number of defended doctorates at one university only were impressive. In 1857, at the Cracow Scientific Society, the Balneological Commission was established by the initiative of Prof. Józef Dietl (1804–1878). In 1905, it was later transformed into the Polish Balneological Society. In 1858, a well-known Jewish businessman, banker and philanthropist from Warsaw, a great supporter of the idea of the development of national health resorts, a friend of Prof. Dietl, Leopold Kronnenberg (1812–1878), initiated the establishment of the National Health Resort Company. It was the body that set up the wide range of basics for organization for all health resorts in Galicia [13]. In 1893, the faculty of medicine was established at the University of Lvov, where students were educated to become physicians employed in, among others, health resorts. Prof. Adolf (Chaim) Abraham Beck (1863–1942) was the rector of this university between 1912 and 1913.

He was considered to be the founder of the Lvov school of physiology and a person who contributed to the development of borderland health resorts. Professor Beck was born in Cracow to a family of Jewish bakers, Szaja Beck and Gustawa née Müller. He graduated from the Middle School of Saint Jacek in his hometown and he studied medicine at the Jagiellonian University (Kraków, Poland). After 1886, he worked at the Department of Physiology and Histology of this university. He defended his doctoral thesis in 1890 and obtained a habilitation in 1894. After the habilitation, he was employed at the University of Lvov, where in 1904–1905 and 1916–1917 he was the dean of the Faculty of Medicine. Professor Beck was a neurologist, physiologist, co-discoverer of brain action currents (1890) and a pioneer of electroencephalography. He carried out studies on the influence of radium on the body which were of great importance for health resort treatment implemented in the early 20^th^ century in radon waters and emanatories [14]. Similarly to Enoch Kisch, he was the editor of the medical journal *Lwowski Tygodnik Lekarski*. It was not a journal with a balneological profile; however, Professor Beck, together with the second editor, Dr. Sieradzki, widely described issues related to health resorts, balneoclimatology and physical medicine. Beck’s university activities supported students of Jewish origin: in 1913, *Biblioteka Słuchaczów*, a medical student organization operating within the structures of this faculty, consisted of 180 participants, of which Jews constituted a great majority [15]. In 1904, the first woman studying medicine in Lvov graduated from this faculty. She was a Jewish woman born in Marijampolė, Maria Matylda Kalmus-Schneiderowa (1879–?) [16]. Professor Beck, as a dean, handed her a graduation diploma [17]. Maria Schneider’s husband, an internist, Nusin Aron Schneider (1873–?) son of Moses, born in Bedrichycze [16], was one of the first graduates of the medical faculty of the Lvov University. He obtained his diploma in 1900 [15]. As a couple, Maria and Nusin Aaron Schneider conducted a joint medical practice in Lvov at 24 Kościuszki Street.

## Jewish doctors and medical innovation in eastern Galicia health resorts

The visible development of physical medicine being the basis of health resort treatment did not take place at universities, where this field was designated as a minor subject for a long time. Wolfgang Krauss believed that physical medicine developed between 1890 and 1914 as a result of the specific situation of Jewish docents, mainly in private practices and laboratories [18]. Apart from the treatments traditionally associated with health resort therapy, which is therefore related to the application of mineral and thermal waters, other treatments appeared, e.g. electrotherapy, fango, electric-hydro baths, modern hydrotherapy, inhalation, sauna, radon treatment and mud treatment. The use of peloids (for example mud) was significant for the development of Galician health resorts. Dr. Hermann Hirschfeld (1825–1885) from Szczecinek (German Neustettin) had a great impact on the popularization of this balneological material in medicine. He examined the therapeutic properties of Kołobrzeg (German Kolberg) mud and contributed to the development of this peloid treatment [19]; however, his greatest contribution was related to balneoclimatic research, which contributed to the development of seaside health resorts. In the nineteenth century, the health resort offer widened to include climate resorts, such as mountain and seaside ones. Until that time, healing baths in sea water were an unknown phenomenon. It can be assumed that the increasing popularity of this kind of resort was also proof of changes in awareness and the upcoming cultural revolution. Hirschfeld, as a chief doctor in the Jewish Sanatorium of Health in Kołobrzeg, conducted extensive research related to the influence of brine, sodium chloride water and sea climate on the body and their important role in health resort therapy [20]. Hirschfeld’s research was continued by another Jewish physician involved in the development of the health resort culture in the borderlands, a microbiologist from the National Institute of Hygiene in Lviv, Prof. Henryk Meisel (1894–1981). He was born into an assimilated Jewish family residing in Przemyśl. He studied medicine at the University of Vienna. From 1922 to 1939 he dealt with, among others, determination of therapeutic properties and treatment suitability of mud from Morszyn. This mud, next to chloride and sodium waters, was the most important balneological material in this health resort city [21]. At the late nineteenth and early twentieth centuries, treatment and prevention of a new group of diseases were introduced in health resorts, namely lifestyle diseases along with health education. A neurologist and urologist, Dr. Samuel Edelman (1891–?) was an example of a physician innovator in this field. He graduated in medicine in 1924. His interests included population metabolic disorders, especially diabetes. He supported the introduction of dietetic nutrition to health resort treatment in Poland as a necessary pillar of this therapeutic process. He worked in Truskawiec, among others in the Badiana and Arkadia villas [22]. His relative, Dr. Adolf Edelman (1883–1944) was professionally related to Vienna, Karlovy Vary and Truskawiec [23]. He was born in Działoszyce near Kielce within the borders of the former Kingdom of Poland. After graduating in medicine at the Jagiellonian University, he practiced among others under the supervision of Dr. Jaworski in Cracow. After defending his PhD in 1911, he began practicing at the Karl Harko von Noorden Clinic (1858–1944) in Vienna. Then, he worked as an assistant and later as a temporary head of the Wilhelminespital internal department at Karel Frederik Wenckebach’s (1864–1939) clinic and as the head doctor of the Children’s Hospital and the Research Institute in Vienna. He carried out studies, among others, on hematology and chemotherapy. In the summer, he examined patients in Karlsbad in the Vulcan villa and sometimes in Truskawiec [24]. He was a medical innovator and a pioneer in health resort treatment, for example related to hematologically based pediatric diseases. There are two medical eponyms related to his name: Edelman syndrome I chronic, acute anemia and Edelman’s syndrome II pancreatitis with hepatocellular infiltration. In 1931 he discovered an element in the blood that he called kinetozyten. Medical treatment of metabolic diseases was also the subject of the study of internist Maximilian Blassberg (1875–1942) from the Jewish hospital in Cracow, who demanded the introduction of “special devices in Polish health resorts for diabetic patients” (translated by Izabela Spielvogel; [25]). His lectures on health resort medicine contributed to the development of this scientific discipline. He was also an active activist for the establishment of the Balneological Institute in Cracow [26]. The Blassberg collaborator, Dr. Adolf Schwarzbart (1882–?), the head of the laryngological department of the Jewish hospital in Cracow, who was involved in the development of inhalation methods in health resort treatment, also contributed to the development of health resort treatment in the borderlands [27]. Inhalation methods were popular, among others, in health resorts in Kosów, Jaremcze, Truskawiec, Druskieniki and Birszrtany. Issues related to the treatment of metabolic diseases at health resorts were also the interest of Dr. Alexander Goldschmiedt (1903–1982) in the 1930s [28]. After the war, he was, among others, a member of the Scientific Council of the Balneological Institute of the Polish Academy of Sciences, employee and rector of the Medical Academy in Łódź (1954–1955) and a chief physician of the health resort in Uzbańsk, Ukraine. In 1956 he moved to Israel. Stefan Kramsztyk (1877–1943) from Warsaw was another well-known physician who was involved in the development of health resort treatment methods. His brother Józef translated the first volume of the famous novel of Thomas Mann’s *The Magic Mountain* into Polish. The novel is set in the Swiss health resort in Davos. Stefan Kramsztyk graduated in medicine from the University of Warsaw in 1903. His scientific works and lectures, also as part of the Polish Society of Balneology and Physical Medicine, contributed to the development of balneotherapy. He was interested in the physicochemical properties of iron-containing mineral waters and their clinical effect on, among others, anemia in children. Together with his wife, he was arrested in 1943. He died in Otwock near Warsaw [29]. Dr. Chaim Blumstein (1890–1946) was another physician involved in the development of the health resort medicine of eastern Galicia. He was a respected surgeon in the Jewish hospital in Grodno and he had a prosperous modernist sanatorium in Druskieniki.

During the war his family was placed in the ghetto in Grodno and Dr. Blumstein was called to the medical service as a physician. Due to the threat of deportation in 1943, the Blumstein family was forced to escape from the ghetto. The family of Janina and Antoni Doch, a physician and friend of Blumstein from before the war, helped them. They hid in a place located 40 km from Grodno, in Staniewicze, at the farm of Edward and Aniela Staniewski. For this purpose, a small room was built in the foundation part of the house. They survived the war after 18 months of hiding in the basement. After the war the Blumsteins came back to Grodno; however, eventually, they settled in Łódź. Unfortunately, Dr. Blumstein did not enjoy his peaceful life for a long time. He died on 6 July 1946. His wife and sons emigrated to Paris. Alexander Blumstein graduated from chemical studies, obtained a PhD in chemistry and together with his wife, Rita Blattberg from Krakow, they left for the USA in 1960 [21].

## Conclusion

On the European level eastern Galicia health resorts were new resorts; their development took place mainly at the late nineteenth and early twentieth century. The Jewish community constitutively contributed to the medical, economic and cultural development of the resorts. It resulted from a wealthy and multilayered Jewish tradition and the integration of Judaism with the hygiene regulations and moral principles of the religion. Eastern Galicia health resorts were local facilities; however, their social function in the region was very important. The model of spending free time that was developed in health resorts was a sign of the upcoming social and custom revolution. In the best period of prosperity, which was at the beginning of the twentieth century, they became an important amenity for the academic and intellectual culture and ethos. The Jews permanently joined this process and co-created the history of the broadly understood health resort culture of the borderlands, and thus the history of Central European culture of life style. How many Jewish physicians and scientists worked in Eastern Galician resorts? It is not possible to obtain any reliable data based on sources and data. The methodology is difficult since, in many cases, their biographical entries are difficult to determine. Many of these physicians were baptized, or sometimes it is impossible to find documents confirming their Jewish origin. Most of the physicians mentioned in the article did not survive the Shoah. They were executed in concentration camps or murdered by the Nazis under unknown circumstances.
